# Differential reconstructed gene interaction networks for deriving toxicity threshold in chemical risk assessment

**DOI:** 10.1186/1471-2105-14-S14-S3

**Published:** 2013-10-09

**Authors:** Yi Yang, Andrew Maxwell, Xiaowei Zhang, Nan Wang, Edward J Perkins, Chaoyang Zhang, Ping Gong

**Affiliations:** 1 School of Computing, University of Southern Mississippi, Hattiesburg, MS 39406, USA; 2 State Key Laboratory of Pollution Control and Resource Reuse & School of the Environment, Nanjing University, Nanjing, China; 3 Environmental Laboratory, US Army Engineer Research and Development Center, Vicksburg, MS 39180, USA; 4 Badger Technical Services, LLC, San Antonio, TX 78216, USA

## Abstract

**Background:**

Pathway alterations reflected as changes in gene expression regulation and gene interaction can result from cellular exposure to toxicants. Such information is often used to elucidate toxicological modes of action. From a risk assessment perspective, alterations in biological pathways are a rich resource for setting toxicant thresholds, which may be more sensitive and mechanism-informed than traditional toxicity endpoints. Here we developed a novel differential networks (DNs) approach to connect pathway perturbation with toxicity threshold setting.

**Methods:**

Our DNs approach consists of 6 steps: time-series gene expression data collection, identification of altered genes, gene interaction network reconstruction, differential edge inference, mapping of genes with differential edges to pathways, and establishment of causal relationships between chemical concentration and perturbed pathways. A one-sample Gaussian process model and a linear regression model were used to identify genes that exhibited significant profile changes across an entire time course and between treatments, respectively. Interaction networks of differentially expressed (DE) genes were reconstructed for different treatments using a state space model and then compared to infer differential edges/interactions. DE genes possessing differential edges were mapped to biological pathways in databases such as KEGG pathways.

**Results:**

Using the DNs approach, we analyzed a time-series *Escherichia coli *live cell gene expression dataset consisting of 4 treatments (control, 10, 100, 1000 mg/L naphthenic acids, NAs) and 18 time points. Through comparison of reconstructed networks and construction of differential networks, 80 genes were identified as DE genes with a significant number of differential edges, and 22 KEGG pathways were altered in a concentration-dependent manner. Some of these pathways were perturbed to a degree as high as 70% even at the lowest exposure concentration, implying a high sensitivity of our DNs approach.

**Conclusions:**

Findings from this proof-of-concept study suggest that our approach has a great potential in providing a novel and sensitive tool for threshold setting in chemical risk assessment. In future work, we plan to analyze more time-series datasets with a full spectrum of concentrations and sufficient replications per treatment. The pathway alteration-derived thresholds will also be compared with those derived from apical endpoints such as cell growth rate.

## Background

Recent advancements in molecular biology technologies, systems biology, and computational toxicology are poised to transform a primarily *in vivo *animal toxicity testing paradigm into a new one dominated by *in vitro *assays [1-3]. This new paradigm makes predictions and cross-species extrapolation based on modes or mechanisms of action (MOAs). However, many challenges remain before this transformation turns into a reality, including: (1) how to incorporate toxicity mechanism information into the next generation risk assessment framework, (2) how to obtain quantitative dose-response and time-course data on the perturbed biological processes or pathways, and (3) how to differentiate transient adaptive perturbations from permanent alterations [1,4-6]. Current MOA approaches mostly focus on identification of differentially expressed genes in canonical pathways. Although some efforts have been made to infer non-observable transcriptomic effect levels (e.g., [7]) or transcriptional benchmark dose values [8], little has been done to investigate gene interaction alterations in toxicity pathways (i.e. pathway perturbations) that are often inferred from time series gene expression profiling data using reverse engineering techniques such as a state-space model with hidden variables [9-12].

To address some of the aforesaid challenges, we conducted a proof-of-concept study using a simple and convenient prokaryotic model organism, *Escherichia coli*, in order to make a direct connection between MOAs and quantitative risk assessment (e.g., toxicity threshold setting) [13,14]. In this study, *E. coli *was exposed to a chemical stressor of three concentrations, and we hypothesized that in stress response: (1) the bacterium had to reassemble biological pathways that differed from their canonical counterparts, and (2) the degree of pathway perturbation was dependent on the exposed concentration. We chose *E. coli *as the test organism also because a microbial live cell reporter array system was constructed recently from its K12 strain MG1655 [15]. This system contained a genome-wide library of modified green fluorescent protein (GFP) expressing promoter reporter vectors. Live Cell Array (LCA) is a novel technology that enables the acquisition of high-resolution time-series profiles of bacterial gene expression by measuring the fluorescence level in living cells carrying fused fluorescent protein [16,17]. This genome-wide *E. coli *LCA has been used to study MOAs of a wide variety of chemicals [18,19] and was used in our study to collect time-course gene expression data [20].

Here, we report a novel differential networks (DNs) approach we developed to derive a toxicity threshold based on the degree of perturbations in reconstructed gene interaction networks. Our approach consists of the following six steps: (1) collect time-series gene expression data of test organisms that received different treatments, (2) identify significantly changed genes involved in normal cellular growth and stress response from the gene expression dataset, (3) reconstruct interaction networks of the altered genes under the control and perturbed/treated conditions using reverse engineering techniques, (4) infer differential edges, i.e., interactions gained or lost from the control to the treated, to construct DNs, (5) annotate and map the genes in the DNs to biological pathways and functions, and (6) establish concentration-pathway perturbation causal relationships. Using this approach we made direct connections between treatment dosage and perturbed pathways.

## Methods

### Live cell array (LCA) system

LCA is a new technology that quantitatively measures the real-time gene expression. It is based on the molecular fusion of a reporting gene system to gene promoters from select stress response regulons. Compared with oligonucleotide hybridization-based microarray technology [21], LCAs avoid complex protocols of pre-treatment and high-cost experimental materials, have less interference, and require less testing time [16]. It involves the generation of a large number of strains that contain transcriptional fusions with fast-folding GFPs and monitoring their accumulation under some certain treatments [22]. An advantage of using bacteria as the organism for LCAs is the ease by which they can be genetically engineered to respond by a dose-dependent signal to environmental stimuli [17]. The promoter activity profiles of individual GFP fusions can be obtained at a very high resolution in a microtiter plate format by determining the difference in fluorescence levels at successive time points after the chemical is administered. Promoter activation or suppression can be easily detected by an increase or a decrease in the fluorescence accumulation rate.

### Collection of *E. coli *LCA time-series dataset

A time-series dataset of dynamic gene expression profiling used in this study was collected in a previous study [20] using the genome-wide *E. coli *LCA made up of twenty-one 96-well plates. Among these 2016 wells, 1870 wells were occupied by 1820 GFP strains with promoter genes (some genes having replicates), another 40 wells were filled with strains with two promoterless genes, and the remaining 106 wells were empty. The standard deviations of the expression values of two promoterless genes were later used to correct for background noise in data normalization steps [20]. There was one empty well on each of the first 20 plates and 86 empty wells on the last plate. These empty wells were used to set the cut-offs in the active gene selection step (see below section "Identification of differentially expressed genes"). Optical density (OD) values were measured before treatment. Then the *E. coli *cells received four treatments of a technical mixture of naphthenic acids (NAs, Sigma Aldrich, St. Louis, MO, USA), i.e., 0 (control), 10, 100, or 1000 mg/L of NAs. The fluorescence levels of all 2016 wells were measured every 10 min for three hours, resulting in a dataset of 18 time points. The entire experiment was performed once without any repeat.

### Data pre-processing

The direct estimation of promoter activities from the time-series profile of the fluorescence level change contains high levels of noise [22]. Therefore, a series of pre-processing procedure needs to be done to correct for noise. First, raw GFP readings were divided by the OD values measured before treatment. OD values reflected the population density of *E. coli *cells in a well before initiation of a treatment. Because the number of cells in each well might be different due to cell growth, by dividing GFP with OD, we got a preliminary value that reflected the activity of our target genes. Then, the result matrix was smoothed by calculating the moving average of every neighbouring three time points. A possible low level of auto-fluorescence of *E. coli *might bring some background noise. To eliminate this type of background noise, the GFP expression produced by the eight promoterless plasmid values were averaged (two promoterless plasmids at four treatments) and subtracted from the values of each gene at the corresponding time point in both experimental and control tests.

Because the promoter activity of each gene might be different at the onset of the experiment, the values of the same gene at time point one in four treatments were averaged, and the differences between the averages and each of the 4 values were calculated. Then, the differences were subtracted from the values of each gene at all of the subsequent time points to eliminate the internal measurement noise. In order to filter the system noise, any value was set to zero if it was less than twice the amount of the standard deviation of the aforementioned processed promoterless values.

### Identification of differentially expressed (DE) genes

DE genes are often sought in genomic studies as they are potential candidates of biomarkers. Different from studies where gene expression is measured at a single time point, time-series experiments have two types of DE genes: Type I: active genes that display differential expression within the same treatment across different time points over the entire course of experiment, and Type II: DE genes whose expression vary significantly between different treatments at any given time point. In this study, we identified the first type of DE genes (active genes) using a one-sample Gaussian Process (GP) regression method developed by [23]. In the GP regression model, the continuous trajectory estimation of a gene expression was treated as an interpolation problem on functions of one dimension, given the observations (gene expression time-series). Subsequently, the differential expression of the gene's profile was assigned a marginal log-likelihood ratio by which it was ranked. In the ranking list, the wells with no *E. coli *cells (empty wells) served as cutoff points. Those genes that ranked higher than the highest ranked empty wells in any of the four treatments were included in the active gene list.

The second type of DE genes was identified in the previous study [20] by applying a linear regression model and a cutoff of 1.5-fold change in gene expression at one or more time points between the control and at least one of the three concentrations. These two types of DE genes were pooled together to form the final list of DE genes.

### Reconstruction of gene interaction networks

An in-house developed Bayesian Learning and Optimization Model (BLOM) was used to reconstruct a network of interaction between the identified DE genes for each of the four treatments. BLOM was based on the state space model with hidden variables and an expectation-maximization algorithm to estimate model parameters (see [9,12] for details). Pre-processed expression data of the identified DE genes were used as the input for BLOM. Like other reverse engineering models such as Dynamic Bayesian Network (DBN) and Probabilistic Boolean Network (PBN), the outcome of BLOM-reconstructed networks is an N × N matrix of connectivity (edge) between two genes with N being the number of nodes/genes. The connectivity is expressed as confidence level in the form of direction (inward, outward and self-to-self), action type (stimulatory if a positive confidence level value, or inhibitive if a negative confidence level value) and strength (absolute confidence level value). The reconstructed networks were visualized using Cytoscape v.2.8.3 [24].

### Inference of differential edges to build differential networks

The reconstructed networks of all three chemical treatments (low, mid and high concentrations of NAs) were compared pair-wise with that of the control to derive differential edges, i.e., edges lost or gained from the control to the chemically treated. From the comparison, the following statistics were obtained for each DE gene: total number of edges in each of the four networks, number of gained or lost edges in the treatment networks, and the percentage of edges changed in the treatment networks. Lost edges are those present in the control network but absent in the low, mid, or high concentration network, whereas gained edges are those absent in the control network but present in the chemical treatment network. The following formula was used to calculate the percentage of edges changed as a result of chemical exposure: (number of gained edges + number of lost edges)/(total number of edges in both the control and the exposure networks). The changed edges (lost or gained) of all involved DE genes were used to construct differential networks for the three chemical treatments.

### Functional annotation and pathway mapping of altered genes

Gene Ontology (GO) terms provide information on molecular function, biological processes and cellular component of a gene product. One gene may have multiple GO terms associated with it. The GO tool at http://www.ecogene.org was used to assign GO terms to the genes of interest (e.g., altered genes). For pathway mapping, we searched the EcoCyc [25], KEGG pathway [26] and RegulonDB [27] databases. The Pathway Tools software v.15.5 [28] was used to extract pathway mapping information from the Ecocyc database.

## Results

### Identified DE genes

DE genes were statistically identified from the time-series gene expression dataset collected using the genome-wide *E. coli *LCA. A total of 47, 11, 45, and 101 genes were identified as active genes (type I DE genes) for the control, low, mid, and high concentrations of NAs exposure respectively, using the GP regression method (Additional file 1). After excluding duplicated genes, there were 111 unique active genes. These genes were pooled together with a group of 85 type II DE genes previously identified using a linear regression model and a 1.5-fold gene expression change filter [20]. This resulted in a final list of 176 unique DE genes, with 20 genes appearing in both type I and type II DE gene lists (Additional file 1).

### Reconstructed interaction networks of DE genes

**Figure 1:**
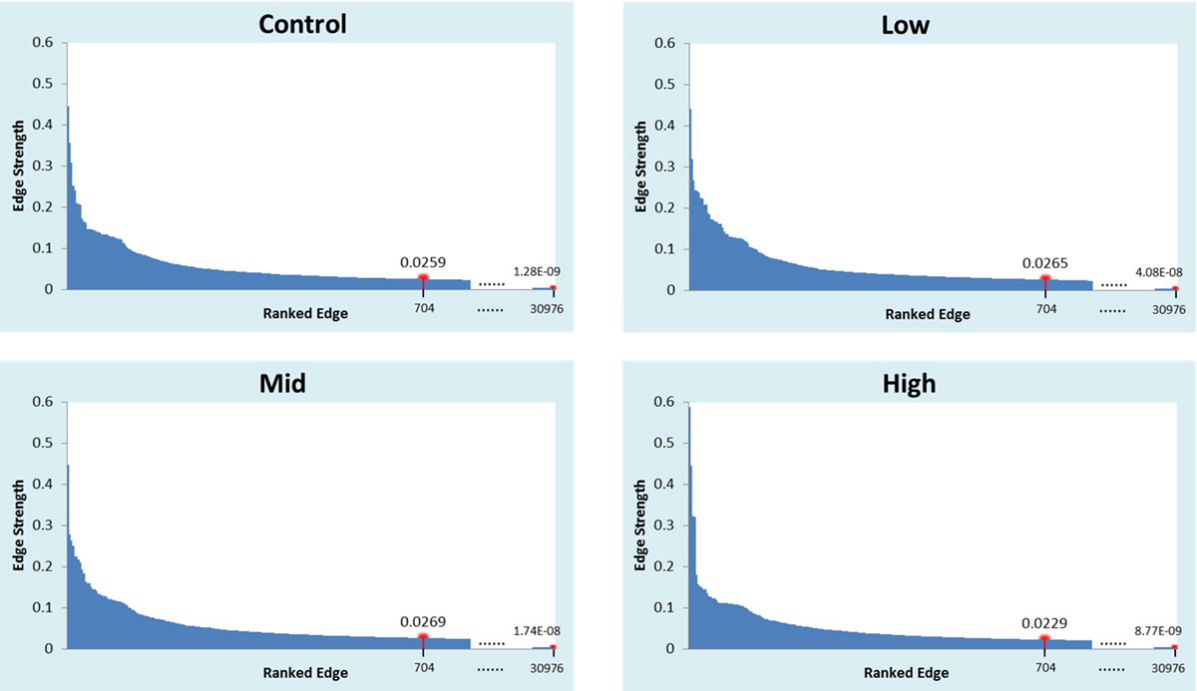
**Histograms of edge strength (absolute values of BLOM-inferred confidence level) distribution for 30976 edges (gene connectivity) in four 176-node networks**. All edges in each network are sorted by their strength and shown on the X-axis in a descending order. Also shown is the strength of the 704^th ^and the lowest ranked edges.

Four interaction networks of 176 nodes (DE genes) were reconstructed using BLOM, one for each treatment. Each network had 30976 (176 × 176) edges, which were ranked by their strength (i.e., absolute value of confidence level) (Additional file 2). Obviously, the ~31K edges are not equally important and should not be treated equally. The higher an edge ranks, the more likely it actually exists. In selecting edges for further analysis, a cut-off level can be set for either the total number of top edges per network or the lowest edge strength allowable in a network. The latest release of EcoCyc database (v. 17.1 as of June 2013) curated 2232 reactions catalyzed by 1500 enzymes that are encoded by 4509 *E. coli *transcription units (genes), suggesting that the average number of interactions per gene might be very low. We have also observed that the total number of edges in a real-world gene interaction network (e.g., KEGG pathways) generally does not exceed four times the total number of its nodes (genes). Furthermore, we plotted four histograms to show edge strength against edge rank (Figure 1). In the four reconstructed networks, the top ranked 704 edges (4 × 176, or 2% of all possible edges) accounted for ~30% of the total strength of all ~31K edges, and the edge strength declined by 94% from the 1^st ^ranked edge to the 704^th ^edge (Additional file 2). Therefore, we selected the top 704 edges per network for further differential edges inference and differential network construction.

### Differential edges and differential networks

**Figure 2:**
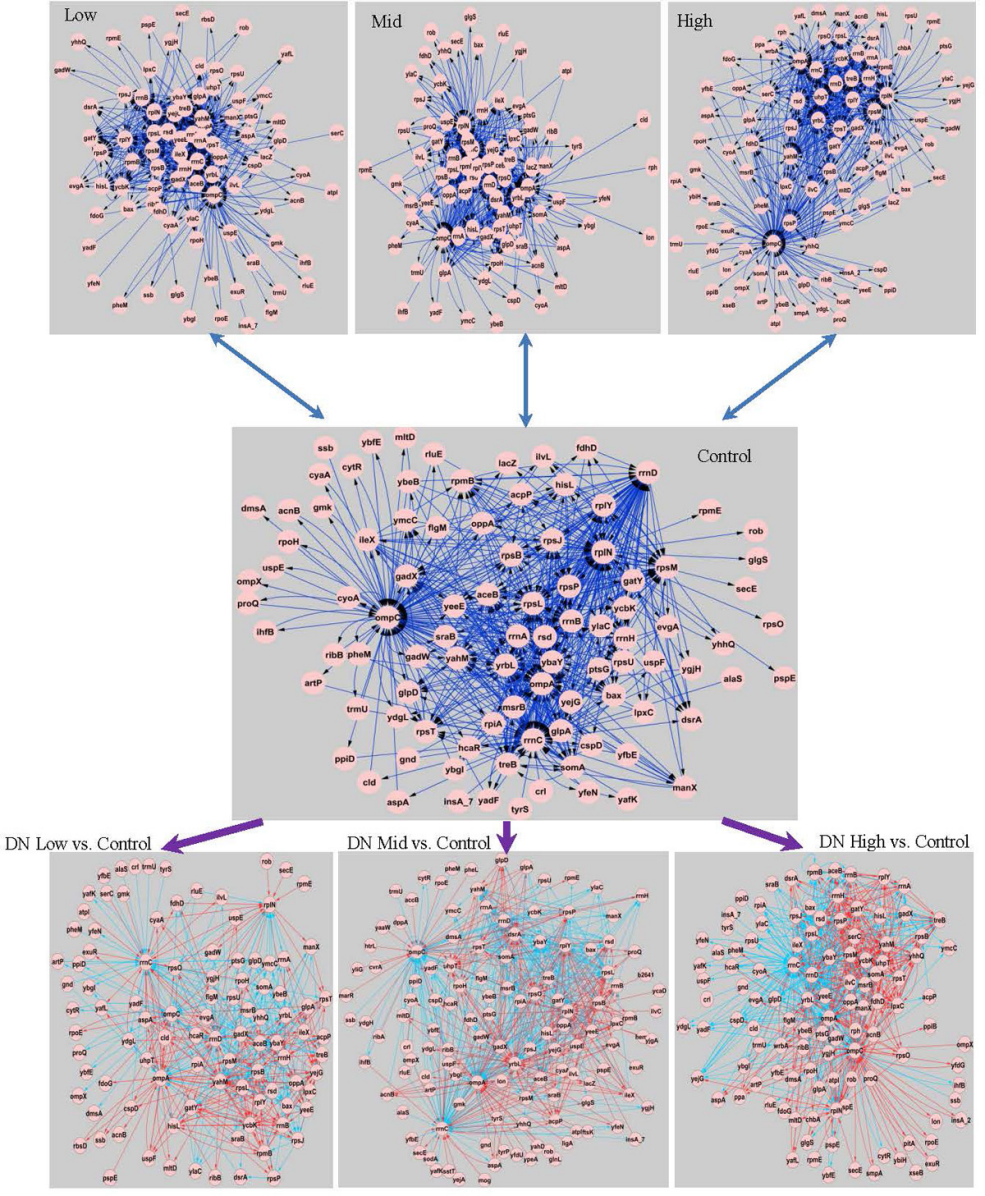
**Differential networks (DNs) obtained by comparing pair-wise the networks reconstructed for three chemical treatments to that of the control treatment**. Each of the four reconstructed networks contains 704 edges. In the DNs, red lines represent gained edges (edges absent in the control network but present in the chemical treatment network), whereas blue lines represent lost edges (edges present in the control network but absent in the chemical treatment network).

Figure 2 presents both the four 704-edge reconstructed networks and the three differential networks. The four reconstructed networks have 96 (control), 87 (low), 82 (mid), and 99 (high) interconnected nodes/genes, with a total of 117 non-redundant DE genes appearing in these networks (Additional file 3). The differential networks were made up of differential edges, i.e., lost and gained edges from the control to a chemical treatment. The number of lost or gained edges were 246 (35% of 704 edges), 299 (42%), and 365 (52%) for the low, mid and high concentration networks, respectively, suggesting a dose-dependence for differential edges. By applying an arbitrary cut-off of 4 differential edges per gene in any one of the differential networks, we removed 37 additional genes and kept the 80 remaining genes for further downstream analysis (see Additional file 3 for statistics on differential edges per DE gene).

### Linking pathway alteration to toxicity threshold

The 80 genes possessing a significant number of differential edges were mapped to biological pathways curated in KEGG and EcoCyc databases as well as to GO terms (see Additional file 4). All but 38 genes were mapped to 35 KEGG pathways. To link pathway alterations to toxicity thresholds, we determined the pathway perturbation degree as the average percentage of edge change per gene for all DE genes involved in any particular KEGG pathway at each exposure concentration (see Additional file 5). A toxicity threshold was defined herein as the concentration at which no pathways were perturbed relative to controls.

The limited number of concentrations that *E. coli *cells were exposed to and the lack of independent treatment replications in the current study prevented a statistical approach to deriving toxicity thresholds from pathway perturbation degrees. Therefore, for purposes of proof of concept, we established a simplified causal relationship between concentration and pathway perturbation, which was defined as the perturbation degree at the high concentration being higher than that at both the mid and the low concentrations. Twenty-two perturbed pathways met this definition (Table 1). These pathways varied substantially in the size of identified DE genes, from one gene in pyruvate metabolism to 16 genes in ribosome. The perturbation degree varied from 0 (i.e., no edge change of the DE genes at all, such as oxidative phosphorylation at both low and mid concentrations) to 1 (i.e., all edges of the DE genes have changed, such as propanoate metabolism at the high concentration).

**Table 1:** The degree of pathway perturbation as related to the exposure concentration of naphthenic acids (NAs).

KEGG pathway name (entry)	low	mid	high	Involved genes (total number)
Ribosome (eco03010)	0.32	0.40	0.45	rplN, rplY, rpmB, rpsB, rpsJ, rpsL, rpsM, rpsO, rpsP, rpsT, rpsU, rrnA, rrnB, rrnC, rrnD, rrnH (16)

Metabolic pathways (eco01100)	0.45	0.42	0.69	aceB, acnB, aspA, gatY, ilvC, lpxC, manX, ribB, rpiA, serC (10)

Microbial metabolism in diverse environments (eco01120)	0.59	0.45	0.78	aceB, acnB, manX, rpiA, serC (5)

Biosynthesis of amino acids (eco01230)	0.55	0.38	0.95	acnB, ilvC, rpiA, serC (4)

Biosynthesis of secondary metabolites (eco01110)	0.55	0.63	0.95	acnB, ilvC, rpiA, yfbE (4)

Amino sugar and nucleotide sugar metabolism (eco00520)	0.61	0.52	0.79	manX, ptsG, yfbE (3)

2-Oxocarboxylic acid metabolism (eco01210)	0.10	0.25	1.00	acnB, ilvC (2)

Aminoacyl-tRNA biosynthesis (eco00970)	0.71	0.67	1.00	ileX, tyrS (2)

Glycerophospholipid metabolism (eco00564)	0.39	0.37	0.76	glpA, glpD (2)

Glyoxylate and dicarboxylate metabolism (eco00630)	0.30	0.49	0.79	aceB, acnB (2)

Nitrogen metabolism (eco00910)	0.68	0.73	0.80	aspA, yadF (2)

Phosphotransferase system (PTS) (eco02060)	0.41	0.37	0.62	ptsG, treB (2)

ABC transporters (eco02010)	0.48	0.50	0.67	oppA (1)

Citrate cycle (TCA cycle) (eco00020)	0.20	0.50	1.00	acnB (1)

Fructose and mannose metabolism (eco00051)	0.33	0.24	0.52	manX (1)

Glycolysis/Gluconeogenesis (eco00010)	0.50	0.33	0.83	ptsG (1)

Lipopolysaccharide biosynthesis (eco00540)	0.17	0.38	0.73	lpxC (1)

Oxidative phosphorylation (eco00190)	0.00	0.00	1.00	ppa (1)

Pantothenate and CoA biosynthesis (eco00770)	0.00	0.00	1.00	ilvC (1)

Propanoate metabolism (eco00640)	0.20	0.50	1.00	acnB (1)

Pyruvate metabolism (eco00620)	0.41	0.49	0.59	aceB (1)

Valine, leucine and isoleucine biosynthesis (eco00290)	0.00	0.00	1.00	ilvC (1)

Twenty-two KEGG pathways were identified as being altered in a concentration-dependent manner by exposure to NAs (low = 10 mg/l, mid = 100 mg/l, high = 1000 mg/l). The perturbation degree was determined as the average percentage edge change per gene for all genes involved in a particular pathway, and concentration-dependence was defined as high > mid and high > low in perturbation degree. Note that perturbation degrees are expressed in decimals instead of percentages.

Even at the low chemical concentration, some pathways were altered to a degree of 50% to 70%, including biosynthesis of amino acids, secondary metabolites, and aminoacyl-tRNA, as well as nitrogen, amino sugar and nucleotide sugar metabolism (Table 1 and Additional file 5). Therefore, the toxicity threshold for NAs would be considered lower than the lowest exposure concentration, 10 mg/L. While compensatory responses in metabolism or biosynthesis may occur at low chemical concentrations, this suggests that pathway perturbations can be a sensitive endpoint for toxicity if additional evidence links the perturbed pathways to adverse outcomes at the physiological, organismal or population level. A more refined toxicity threshold could be derived using regression approaches such as a benchmark dose method [29,30] if more concentrations were tested in addition to more replications per treatment.

## Discussion

Cellular exposure to toxicants often results in pathway alterations reflected as changes in gene expression regulation and gene interactions. Such information is often used to elucidate toxicological modes of action. From a risk assessment perspective, alterations in biological pathways are a rich resource for setting toxicant thresholds, which may be more sensitive and mechanism-informed than traditional toxicity endpoints. Here we reported a proof-of concept study to connect pathway perturbation with toxicity threshold setting using the DNs approach to analyzing time-series gene expression datasets.

Studies involving gene expression profiling at a single time point have limited power in both deciphering MOAs and quantitative risk assessment because the snapshot of gene expression profiling misses the dynamic and interactive nature of cellular gene expression. As the costs of acquiring genome-wide gene expression technologies steadily decrease, it has become more feasible and affordable to perform time-series gene expression studies (see databases such as GEO (Gene Expression Omnibus) at http://www.ncbi.nlm.nih.gov/geo and ArrayExpress at http://www.ebi.ac.uk/arrayexpress/ for large-scale time-series datasets). In order to take advantages of technological advancements in high throughput microarray, DNA sequencing and LCA, novel experimental and computational approaches are needed to transform conventional toxicology to predictive toxicity in order to meet the requirements of more rapidly assessing toxicity of chemicals and other materials to humans and animals in the 21st century risk assessment [1-4,6,14].

A distinction has to be made between edges in the graphical representation of a literature curation-based biological pathway (e.g., KEGG pathway) and those derived *in silico *from time-course data. The former are experimentally validated interactions, whereas the latter represent potential gene-gene interactions. It was not our intention to estimate the accuracy of our BLOM-inferred edges by comparing them with those gene-gene interactions curated in KEGG pathways or EcoCyc databases, but rather to use the inferred edges to provide an estimate of the overall degree of alteration in a gene's interaction/connectivity with other genes.

Our DNs approach differs significantly from existing DE gene-based approaches such as the Gene Set Enrichment Analysis (GSEA) method [31] and the genomic benchmark dose method [29,30] in at least three aspects: (1) our approach is based on reverse engineering techniques including BLOM, PBN and DBN; (2) significantly altered pathways are identified through analysis of DNs instead of enrichment of DE genes mapped to canonical pathways; and (3) our approach is particularly suitable for analyzing time-series gene expression datasets whereas existing approaches like GSEA are suitable for static datasets often collected at a single time point.

While the current proof-of-concept study demonstrates that our DNs approach is capable of identifying perturbed pathways in relation to chemical exposure, several caveats remain to be resolved. First, no analytical chemistry work was carried out in the study to confirm the concentration and/or biotransformation of NAs throughout the 3-hr exposure. Many xenobiotic toxicants such as polycyclic aromatic hydrocarbons, aryl and heterocyclic amines require metabolic activation by cytochrome P450 metabolism [32]. Chemical analysis, in parallel to bioassays, can provide useful information about the chemical(s) of concern for toxicity threshold derivation. Second, as the time-course dataset used in this study lacks treatment replication, the derived degrees of pathway perturbation (Table 2) are statistically inadequate to determine a point of departure or toxicity threshold for each perturbed pathway. This limitation can be ameliorated by incorporating more treatments and treatment replications into the experimental design. Third, no apical endpoints such as physiology or biochemistry assays were measured, which would have permitted linkage of the derived lowest observable pathway perturbation concentrations to toxicity thresholds derived from apical endpoints and hence applicable to chemical risk assessment [8,33]. Finally, while the *E. coli *LCA system is a very rapid approach to assessing impacts on biological pathways, it is not free from limitations. For instance, less than 50% of known transcriptional genes have a promoter that can be fused with a GFP [15], leading to an incomplete genome coverage. Alternative high-throughput technologies such as DNA microarray and next-generation sequencing can be used to generate genome-wide time-series gene expression dataset.

## Conclusions

Findings from this proof-of-concept study suggest that our approach has a great potential in providing a novel and sensitive tool for threshold setting in chemical risk assessment. In future work, we plan to analyze more time-series datasets with a full spectrum of concentrations and sufficient replications per treatment, and eventually extrapolate our approach from prokaryotic systems to eukaryotes. The pathway alteration-derived thresholds will also be compared with those derived from apical toxicology, biochemistry and physiology endpoints such as cell growth rate.

## Competing interests

The authors declare that they have no competing interests.

## Authors' contributions

PG conceived the project and designed the approach. YY conducted gene selection, network reconstruction and comparison, and gene functional annotation. XZ provided the live cell experimental data. XZ and AM participated in data pre-processing and analysis. PG, CZ and NW supervised this work. YY, PG and AM drafted the manuscript. PG, EJP, XZ, CZ and NW revised the manuscript. All authors read and approved the final manuscript.

## Supplementary Material

Additional file 1 **Differentially expressed genes**. Breakdown of the two types of differentially expressed genes Click here for file

Additional file 2 **Gene connectivity**. Connectivity between 176 genes in four networks reconstructed from the control *E. coli *cells and those treated with 10, 100 and 1000 mg/L naphthenic acids Click here for file

Additional file 3 **Differential edges**. Statistics on differential edges derived by pair-wise comparison between the top 704 edges of the four reconstructed networks (control vs. low/mid/high) Click here for file

Additional file 4 **Pathway mapping**. Annotation, GO terms and pathway mapping to EcoCyc and KEGG pathway databases for 80 select genes with at least 4 differential edges in any of the three differential networks Click here for file

Additional file 5 **Pathway perturbation**. Pathway perturbation was determined as the average percentage in edge change per gene of all genes involved in a particular pathway. Click here for file

## Declarations

Funding for publication of the article came from NSF EPSCoR (NSF #EPS-0903787).

This article has been published as part of *BMC Bioinformatics *Volume 14 Supplement 14, 2013: Proceedings of the Tenth Annual MCBIOS Conference. Discovery in a sea of data. The full contents of the supplement are available online at http://www.biomedcentral.com/bmcbioinformatics/supplements/14/S14.

## References

[B1] Bhattacharya S Zhang Q Carmichael PL Boekelheide K Andersen ME Toxicity testing in the 21 century: defining new risk assessment approaches based on perturbation of intracellular toxicity pathways PLoS One 2011 14 e20887 10.1371/journal.pone.0020887 21701582 PMC3118802

[B2] Collins FS Gray GM Bucher JR Toxicology. Transforming environmental health protection Science 2008 14 906 907 10.1126/science.1154619 18276874 PMC2679521

[B3] Krewski D Acosta D Jr Andersen M Anderson H Bailar JC Boekelheide K Toxicity testing in the 21st century: a vision and a strategy J Toxicol Environ Health B Crit Rev 2010 14 51 138 10.1080/10937404.2010.483176 20574894 PMC4410863

[B4] Cote I Anastas PT Birnbaum LS Clark RM Dix DJ Edwards SW Advancing the next generation of health risk assessment Environ Health Perspect 2012 14 1499 1502 10.1289/ehp.1104870 22875311 PMC3556615

[B5] Edwards SW Preston RJ Systems biology and mode of action based risk assessment Toxicol Sci 2008 14 312 318 10.1093/toxsci/kfn190 18791183

[B6] Tannenbaum LV Is NexGen really the next generation of risk assessment? Integr Environ Assess Manag 2012 14 213 214 10.1002/ieam.1297 22431348

[B7] Ludwig S Tinwell H Schorsch F Cavaille C Pallardy M Rouquie D A molecular and phenotypic integrative approach to identify a no-effect dose level for antiandrogen-induced testicular toxicity Toxicol Sci 2011 14 52 63 10.1093/toxsci/kfr099 21525395

[B8] Thomas RS Clewell HJ Allen BC Yang L Healy E Andersen ME Integrating pathway-based transcriptomic data into quantitative chemical risk assessment: a five chemical case study Mutat Res 2012 14 135 143 10.1016/j.mrgentox.2012.01.007 22305970

[B9] Li P Inferring Gene Regulatory Networks from Time Series Microarray Data 2009 Hattiesburg, MS: University of Southern Mississippi PhD Dissertation

[B10] Li Z Shaw SM Yedwabnick MJ Chan C Using a state-space model with hidden variables to infer transcription factor activities Bioinformatics 2006 14 747 754 10.1093/bioinformatics/btk034 16403793

[B11] Rangel C Angus J Ghahramani Z Lioumi M Sotheran E Gaiba A Modeling T-cell activation using gene expression profiling and state-space models Bioinformatics 2004 14 1361 1372 10.1093/bioinformatics/bth093 14962938

[B12] Wu X Li P Wang N Gong P Perkins EJ Deng Y State Space Model with hidden variables for reconstruction of gene regulatory networks BMC Syst Biol 2011 14 Suppl 3 S3 10.1186/1752-0509-5-S3-S3 22784622 PMC3287571

[B13] Ben-Israel O Ben-Israel H Ulitzur S Identification and quantification of toxic chemicals by use of Escherichia coli carrying lux genes fused to stress promoters Appl Environ Microbiol 1998 14 4346 4352 9797288 10.1128/aem.64.11.4346-4352.1998 PMC106650

[B14] Currie RA Toxicogenomics: the challenges and opportunities to identify biomarkers, signatures and thresholds to support mode-of-action Mutat Res 2012 14 97 103 10.1016/j.mrgentox.2012.03.002 22445948

[B15] Zaslaver A Bren A Ronen M Itzkovitz S Kikoin I Shavit S A comprehensive library of fluorescent transcriptional reporters for Escherichia coli Nat Methods 2006 14 623 628 10.1038/nmeth895 16862137

[B16] Elad T Lee JH Belkin S Gu MB Microbial whole-cell arrays Microb Biotechnol 2008 14 137 148 10.1111/j.1751-7915.2007.00021.x 21261831 PMC3864447

[B17] Melamed S Elad T Belkin S Microbial sensor cell arrays Curr Opin Biotechnol 2012 14 2 8 10.1016/j.copbio.2011.11.024 22176747

[B18] Gou N Gu AZ A new Transcriptional Effect Level Index (TELI) for toxicogenomics-based toxicity assessment Environ Sci Technol 2011 14 5410 5417 10.1021/es200455p 21612275

[B19] Su G Zhang X Liu H Giesy JP Lam MH Lam PK Toxicogenomic mechanisms of 6-HO-BDE-47, 6-MeO-BDE-47, and BDE-47 in E. coli Environ Sci Technol 2012 14 1185 1191 10.1021/es203212w 22111525

[B20] Zhang X Wiseman S Yu H Liu H Giesy JP Hecker M Assessing the toxicity of naphthenic acids using a microbial genome wide live cell reporter array system Environ Sci Technol 2011 14 1984 1991 10.1021/es1032579 21309546

[B21] Ehrenreich A DNA microarray technology for the microbiologist: an overview Appl Microbiol Biotechnol 2006 14 255 273 10.1007/s00253-006-0584-2 17043830

[B22] Aichaoui L Jules M Le CL Aymerich S Fromion V Goelzer A BasyLiCA: a tool for automatic processing of a Bacterial Live Cell Array Bioinformatics 2012 14 2705 2706 10.1093/bioinformatics/bts422 22764159

[B23] Kalaitzis AA Lawrence ND A simple approach to ranking differentially expressed gene expression time courses through Gaussian process regression BMC Bioinformatics 2011 14 180 10.1186/1471-2105-12-180 21599902 PMC3116489

[B24] Smoot ME Ono K Ruscheinski J Wang PL Ideker T Cytoscape 2.8: new features for data integration and network visualization Bioinformatics 2011 14 431 432 10.1093/bioinformatics/btq675 21149340 PMC3031041

[B25] Karp PD Riley M Saier M Paulsen IT Collado-Vides J Paley SM The EcoCyc Database Nucleic Acids Res 2002 14 56 58 10.1093/nar/30.1.56 11752253 PMC99147

[B26] Altman T Travers M Kothari A Caspi R Karp PD A systematic comparison of the MetaCyc and KEGG pathway databases BMC Bioinformatics 2013 14 112 10.1186/1471-2105-14-112 23530693 PMC3665663

[B27] Salgado H Peralta-Gil M Gama-Castro S Santos-Zavaleta A Muniz-Rascado L Garcia-Sotelo JS RegulonDB v8.0: omics data sets, evolutionary conservation, regulatory phrases, cross-validated gold standards and more Nucleic Acids Res 2013 14 D203 D213 10.1093/nar/gks1201 23203884 PMC3531196

[B28] Karp PD Paley S Romero P The Pathway Tools software Bioinformatics 2002 14 Suppl 1 S225 S232 10.1093/bioinformatics/18.suppl_1.S225 12169551

[B29] Thomas RS Allen BC Nong A Yang L Bermudez E Clewell HJ III A method to integrate benchmark dose estimates with genomic data to assess the functional effects of chemical exposure Toxicol Sci 2007 14 240 248 10.1093/toxsci/kfm092 17449896

[B30] Yang L Allen BC Thomas RS BMDExpress: a software tool for the benchmark dose analyses of genomic data BMC Genomics 2007 14 387 10.1186/1471-2105-8-387 17961223 PMC2198920

[B31] Subramanian A Tamayo P Mootha VK Mukherjee S Ebert BL Gillette MA Gene set enrichment analysis: a knowledge-based approach for interpreting genome-wide expression profiles Proc Natl Acad Sci USA 2005 14 15545 15550 10.1073/pnas.0506580102 16199517 PMC1239896

[B32] Shimada T Murayama N Yamazaki H Tanaka K Takenaka S Komori M Metabolic Activation of Polycyclic Aromatic Hydrocarbons and Aryl and Heterocyclic Amines by Human Cytochromes P450 2A13 and 2A6 Chem Res Toxicol 2013 10.1021/tx3004906 PMC3713097 23432465

[B33] Thomas RS Wesselkamper SC Wang NC Zhao QJ Petersen DD Lambert JC Temporal concordance between apical and transcriptional points of departure for chemical risk assessment Toxicol Sci 2013 14 180 194 10.1093/toxsci/kft094 23596260

